# Optical absorbing origin of chiroptical activity in planar plasmonic metasurfaces

**DOI:** 10.1038/s41598-017-10532-6

**Published:** 2017-08-31

**Authors:** Atefeh Fazel Najafabadi, Tavakol Pakizeh

**Affiliations:** Faculty of Electrical Engineering, K. N. Toosi University of Technology, Tehran, 1631714191 Iran

## Abstract

As a significant characteristic of many biomolecules, chemical substances, and artificial nanostructures, chirality conduce different types of optical interactions with the spin angular momentum of the impinging light field. Although, chiral arrangement and spatial phase retardation are the key factors for obtaining chirality in three-dimensional (3D) structures, the origin of chirality in the feasible planar structures has not been thoroughly addressed. Here using an intuitive and simple analytical approach, called cross-hybridization model, the essence and properties of the optical chirality of individual planar nanostructures are unveiled. In order to fundamentally address this chirality in terms of circular dichroism (CD), the chiroptical response of a simple dimer composed of the lossy nanoblocks in L-shape arrangement are investigated based on the provided optical interaction and loss effects. The theoretical findings, adequately supported by the numerical calculations, reveal that chiroptical activity occurs predominantly due to handedness-dependent absorption or heating loss in a nanostructured metasurface.

## Introduction

Chiroptical properties of non-superimposable biological molecules and chiral nanostructures on their mirror images, have gained tremendous interest in recent years. Biomolecules mainly show sensitivity to one of the left or right circular polarized light. This results in the investigation the tendency of the biomolecules and construction drugs with an efficient enantiomer^1^. Similar to natural materials, well organized artificial metasurfaces indicate different behavior to the spin angular momentum of the light which arises unlike refractive indices and absorption rates for left and right circularly polarized (LCP and RCP, respectively) waves^1–7^. Therefore, novel photonic devices such as circular dichroism metamirrors, optical detectors for Stokes parameters’ polarimetry, chiral sensors, and metamaterials for terahertz nonlinear optics and quantum information processing are suggested^6–12^.

However, breaking symmetry in 3D nanostructures is a necessary and sufficient condition for obtaining chirality^5, 10, 13, 14^, the physical origin of the chirality in planar nanostructures not yet entirely addressed. Recently using a novel developed suit, it has been experimentally shown that CD –the difference in optical properties of LCP and RCP light– in planar metasurfaces occurs due to handedness dependent of ohmic heating^15^. Moreover, it has been numerically demonstrated that homogenous structures composed of silicon nanodisks do not posse chirality^16^.

Here, the electromagnetic interactions between the simplest planar chiral nanostructure and different spin angular momentum of the incident light are modeled using cross coupled dipoles in quasistatic regime. A dimer of nanoparticles in L-shape arrangement, which might be called meta-atom of an arbitrary metasurface, is considered. This simple building-block conveys interesting and unique optical properties in terms of optical CD, granted by the properly excited and hybridized localized surface plasmon resonances (LSPRs). It is reported that absorption or loss is the major root of chirality in planar metasurfaces composed of optically interacting nanoparticles as their building blocks. Although a planar lossy nanostructure which is non-superimposable on its mirror image show substantially different absorption under the RCP and LCP light illuminations, in a drastic contrast, a lossless one is achiral even its comprising nanoparticles are arranged in a handedness order.

## Results and Discussions

In order to theoretically analyze the chiroptical properties of a system composed of nanoparticles, one should first model the nanostructure precisely and derives the optical properties such as absorption (C_abs_), scattering (C_sca_), and extinction (C_ext_) cross-sections. If the size of the nanoparticles is much smaller than the wavelength, modeling each particle with an electric point dipole is satisfactory. This modeling yields the optical properties of the whole structure by solving the coupled-dipole equations (CDE)^17^. For an L-shape dimer composed of two nanoparticles with the general properties, the CDE expressed in Eq. (1).

1 $${P}_{{\rm{i}}}-{\alpha }_{{\rm{i}}}{A}_{ij}{P}_{{\rm{j}}}={\alpha }_{{\rm{i}}}{E}_{\text{inc},i}$$

where ${\alpha }_{i}$(*i, j* = *x*, *y*) is the electric polarizability of a single nanoparticle^18, 19^, *A*_ij_ is the cross-coupling term between two nanoparticles, *P*_j_ is the amplitude of the induced polarizability vector, and *E*_inc,j_ is the incident electric filed at the location of particle *j*. It is common to nanosystems that absorption is dominant relative to the scattering, C_abs_ approximates the C_ext_. However, for planar nanostructures there is not any difference between C_ext_ of the LCP and RCP waves; therefore for determination of the CD_abs_, the C_abs_ should be calculated with high accuracy^20^. This aim is achievable by calculation C_sca_ or the power radiated by an array of oscillating dipoles, although negligible. Consequently, the absorption C_abs_ can be written as:

2 $${{\rm{C}}}_{{\rm{a}}{\rm{b}}{\rm{s}}}=\frac{4\pi k}{|{{\bf{E}}}_{{\rm{i}}{\rm{n}}{\rm{c}}}{|}^{2}}\sum _{j=x,y}^{}\text{Im}\{{{\bf{P}}}_{{\rm{j}}}.{({\alpha }_{{\rm{j}}}^{-1})}^{\ast }{{{\bf{P}}}_{{\rm{j}}}}^{\ast }\}-\frac{2}{3}{k}^{3}\int d{\rm{\Omega }}{|\sum _{j=x,y}^{}[{{\bf{P}}}_{{\rm{j}}}-\hat{{\bf{n}}}(\hat{{\bf{n}}}.{{\bf{P}}}_{{\rm{j}}})]\exp (-ik\hat{{\bf{n}}}.{{\bf{r}}}_{{\rm{j}}})|}^{2}$$

where *k* is the wave vector, $d{\rm{\Omega }}$ is the element of solid angle, **r**_j_ is the vector in the desired direction of scattering, and $\hat{{\bf{n}}}$ is the corresponding unit vector^21^. After some rigorous but routine algebra (the details are explained in the Method section), the ${{\rm{CD}}}_{abs}={{\rm{CD}}}_{abs}^{LCP}-{{\rm{CD}}}_{abs}^{RCP}$ for a chiral system composed of two nanoparticles is reduced to:

3 $${{\rm{CD}}}_{{\rm{abs}}}=\frac{32\pi }{3}{k}^{4}\,{A}_{{\rm{xy}}}\frac{|{\alpha }_{{\rm{x}}}{|}^{2}\text{Im}\{{\alpha }_{y}\}-{|{\alpha }_{y}|}^{2}\text{Im}\{{\alpha }_{x}\}}{{|1-{A}_{{\rm{xy}}}^{2}{\alpha }_{{\rm{x}}}{\alpha }_{y}|}^{2}}$$

Using this fundamental and simple relation, we can easily trace the origin of the CD in planar nanostructures. Importantly, since the spatial phase retardation, in the propagation direction is zero for the planar structures, CD_ext_ = 0; and as a result and to justify the relation between CD_abs_, CD_sca_, and CD_ext_, CD_sca_ = −CD_abs_ 22. Expectedly, if the nanoparticles are maund by setting the denominatorde of lossless metals or dielectrics, Im{*α*} = 0, so that CD_abs_ vanishes (see Eq. (3)). In this ideal case, the absorption is inherently zero and consequently CD_sca_ = −CD_abs_ = 0, and thus no sign of chirality can be seen in the optical properties. Similarly, it has been recently discussed that handedness nanostructure composed of silicon nanodisks breaks to posse chirality; although, they have interpreted this based on non-induced magnetic dipole moments^16^. The introduced comprehensive formula in here, i.e. Eq. (3), clearly demonstrates that even if the dipole moments are induced in a lossless nanostructure, it remains achiral. Similarly, it makes that obvious to have CD in the planar nanostructures the optical interaction of the nanoparticles, denoted by *A*_*xy*_, which depends on the arrangement and the separation distance of the nanoparticles, should be noticeable. Importantly, these coupling effects of nanoparticles on each other should differ, depending on the polarization. In the regime where the assumptions of the CDE approach are valid, these could be considered as two crucial criteria for designing chiral nanostructures or meta-atoms for the intended optically active planar metasurfaces.

Additionally, the peaks’ positions of the resulting CD_abs_ as well as the elementary absorption, C_abs_ (Eq. 2), of the hybridized LSPR modes, i.e. the in-phase (anti-bonding) and out-of-phase (bonding) modes, are analytically found by setting the denominator in Eq. 3 to zero: 1−(*A*_xy_ *α*)^2^ = 0, by assuming *α*_x_ ≃ *α*_y_ = *α*. Based on this condition, the electric polarizability ($\alpha (\omega )={V}(\varepsilon (\omega )-\,{\varepsilon }_{m}){[{\varepsilon }_{m}+{l}(\varepsilon (\omega )-{\varepsilon }_{m})]}^{-1}$)^20^, and the ideal (lossless) Drude model ($\varepsilon (\omega )={\varepsilon }_{\infty }-{{\omega }_{P}}^{2}/{\omega }^{2}$) for the dielectric function of the nanoparticles^23^, these energies are found:

4 $${\omega }_{\pm }^{2}={{\omega }_{{\rm{p}}}}^{2}/[({\varepsilon }_{\infty }-{\varepsilon }_{{\rm{m}}})\pm {\varepsilon }_{{\rm{m}}}{(V{A}_{{\rm{xy}}}\pm l)}^{-1}]$$

where *ω*_p_ and ${\varepsilon }_{\infty }$ are the Drude parameters, and *V* and *l* are the volume and the static depolarization factor of the nanoparticles or nanoblocks along their major axis.

**Figure 1:**
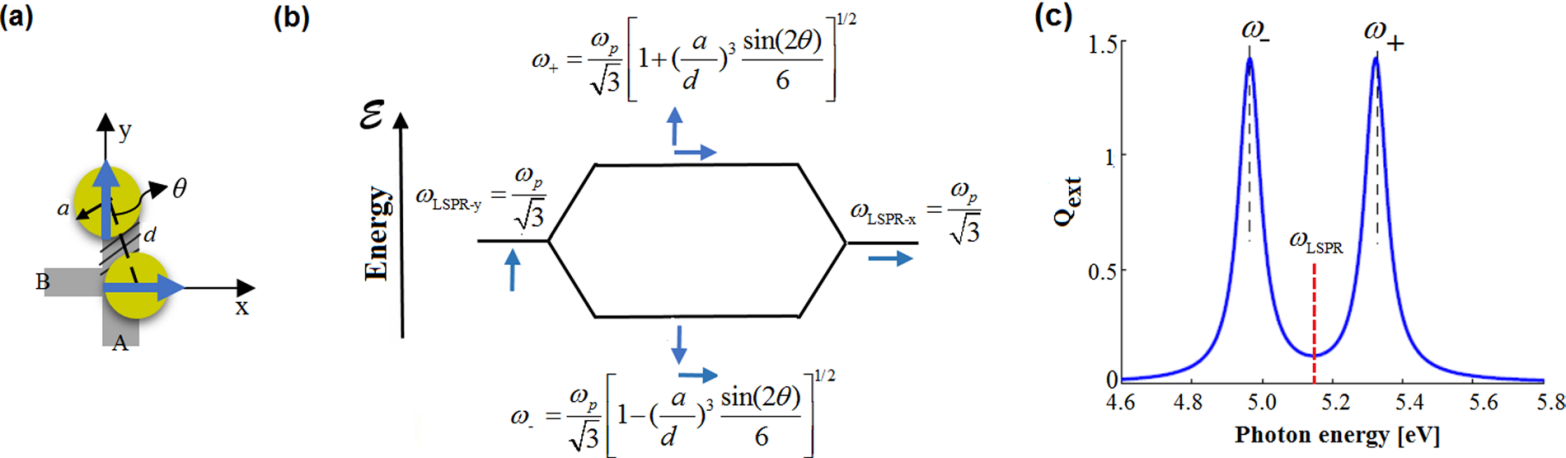
The cross hybridization model and properties. (**a**) Schematic representation of the planar chiral dimer. (**b**) Cross hybridization model of the nanosystem. (**c**) Optical C_ext_ of the Drude dimer presented in (**a**) with *a* = 10 nm, *d* = 25 nm, and *θ* = 25°. The resonance frequencies of a single particle (*ω*_LSPR_) and interacting dimer (*ω*_±_) are denoted by the dotted vertical lines.

In the most trivial case, a schematic view of the L-shape dimer composed of two nanospheres with the diameter 2*a* and the interparticle spacing *d* is illustrated in Fig. 1(a). There, it is acceptable that the vertical finite-sized equivalent dipole in y-direction experiences different surrounding environment (shaded-area A), compared to the nearby horizontal x-dipole (shaded-area B). In this context, since the y-dipole couples more strongly than x-dipole, two separate effective polarizabilities, *α*_y_ and *α*_x_, can be considered; where they can be referred to the slightly different effective-sizes of the considered dipoles. Thus, presumably the polarization-dependent optical cross-coupling of these two dipoles leads to the some sort of CD. Figure 1(b) depicts the cross-hybridization model and the induced dipole moments for the L-shape dimer. This can be compared to the conventional hybridization model where the point-dipoles are co-polarized^24^. Also, the optical extinction efficiency, Q_ext_ of the L-shaped plasmonic nanosystem with *θ* = 25 deg., *d* = 25 and *a* = 10 nm, and described by the Drude model with ${\omega }_{P}$ = 8.915 and *γ* = 0.477 eV, which non-uniformly illuminated by the linearly-polarized incident field, *E*_inc,x_, is depicted in Fig. 1(c) in which the LSPR energy of a single particle (*ω*_LSPR_) and the resulting hybridized energies *ω*_±_ of the considered dimer are determined. Moreover, since the nanoparticles are very close, throughout the paper only the nearfield term in *A*_xy_ is is considered. Thus, based on the arrangement and geometry seen in Fig. 1(a), the term *A*_xy_ = −3sin(2*θ*)/(2 ${d}^{3}$).

In this illustrative case, the relation in Eq. (4) becomes even simpler by assuming the ideal Drude nanospheres with ${\varepsilon }_{\infty }$ = 1, *l* = 1/3, and *V* = *a* ^3^/3, immersed in air (${\varepsilon }_{{\rm{m}}}$=1): ${\omega }_{\pm }={\omega }_{{\rm{LSPR}}}{[1\pm 1/6{(a/d)}^{3}\sin (2\theta )]}^{1/2}$; where ${\omega }_{{\rm{LSPR}}}={\omega }_{{\rm{p}}}/\sqrt{3}$. Remarkably, it can be also affirmed that cross-coupling term diminishes when the nanoparticles are along the same axis, for instance y-axis or *θ* = 0°. Nevertheless, degree of freedom for the resonant point-dipoles’ orientation is infinite in the nanosphere case; and to make this more practical and limited to the chiral case, then one should consider other types of the dipolar LSPRs. Thereby, the nanoblocks, modeled by the prolate nanospheroids, in the same arrangement are employed to reach this goal. It allows only two cross point-dipoles, one on each, to be efficiently excited, in the desired spectral range and upon uniform illuminations. Besides, the optical interaction of these nanoparticles is more pronounced, compared to the nanospheres, which results in substantial polarization-dependent effects. Thus, in the next section, the optical interaction and chiroptical properties of the planar nanostructure composed of nanoblocks are studied in details.

**Figure 2:**
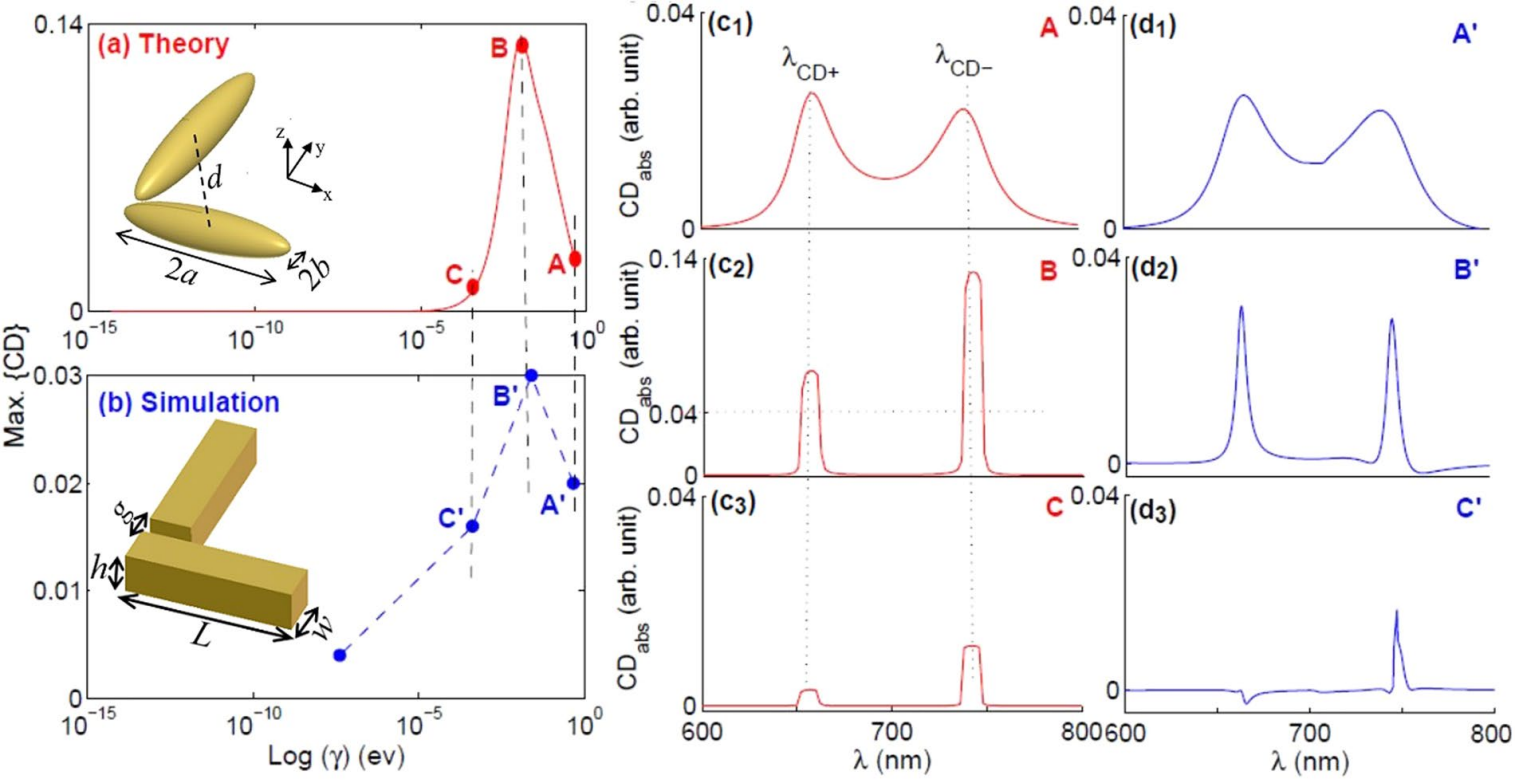
The CD_abs_ trend versus damping-factor γ in a dimer of Drude nanoblocks. Max. {CD_abs_} in terms of γ, theoretically (**a**) and numerically (**b**), are shown. The column (**c**) shows the theoretical CD_abs_ spectra for three specified values of γ, red points at Fig. 1(a). Corresponding numerical results are depicted in the right-column (**d**). The geometric parameters are 2*a* = 80 nm, 2*b* = 17 nm, *d* = 50 nm, *L* = 80 nm, *w* = *h* = 20 nm, and *g* = 10 nm. The vertical black dashed-lines in the c-column denote the fix *λ*_CD±_ of the Au dimer.

Finding the origin of CD in planar nanostructures, we try to omit complexities and different effective parameters as far as possible. Hence, the nanoparticles are chosen small enough that one can ignore the correction term regarding the energy loss due to radiative reaction^18^. Consequently, a dimer of nanoblocks in a handedness arrangement with the length *L*, same width and height *w* = *h*, and equal spacing along the *x* and *y* axes is selected. The geometry of the dimer is depicted in the inset of Fig. 2(b). In the theoretical modeling, each nanoblock is electromagnetically described by the quasistatic polarizability of a prolate nanospheroid (*a* > *b* = *c*), where *a*, *b*, and *c* are the major and minor semiaxis of the spheroid (see inset in Fig. 2(a)). Moreover, the rigorous electromagnetic simulations are performed using the finite integration technique (FIT)^25^. Several cases are studied to thoroughly investigate the role of different parameters in CD_abs_. At first the complex dielectric function is described by the Drude model. Then, real metals with the experimentally tabulated permitivities, as more practical and sensible cases, are studied.

### CD of the Drude planar nanostructures

In the section, we employ a dimer of Drude nanoblocks with *L* = 80 nm, *w* = *h* = 20 nm, and equal gap spacing along the *x*- and *y*-axes, *g* = 10 nm. Accordingly, in the modeling, the nanospheroids with 2*b* = 2*c* = 17 nm, and *d* = 50 nm are considered (see insets in Fig. 2(a,b)). However, for the x-oriented nanospheroid 2*a* = 80 nm and the for other one 82 nm, effectively accounting for stronger interaction about the finite-sized gap area and along y-axis, as previously discussed in terms of *α*_x_ and *α*_y_. The LCP and RCP plane-waves are illuminated to the structure from the top. To easily address the roles of the parameters like crucial absorption in the observed phenomenon, the complex dielectric function is described by the Drude model in which the parameters ${\varepsilon }_{\infty }$,${\omega }_{P}$, and *γ* in order are 9.068, 8.915, and 0.477 eV, partially fitted to the experimental data of gold^23^. Nevertheless, the general trend of CD_abs_ in terms of different losses remains ambiguous. The normalized imaginary-part of *α* of a nanospheroid, based on physical parameters is given by:

5 $$\text{Im}\{\alpha \}=\frac{\varepsilon ^{\prime\prime} {\varepsilon }_{m}}{[{(l\varepsilon ^{\prime} +(1-l){\varepsilon }_{m})}^{2}+{l}^{2}{\varepsilon ^{\prime\prime} }^{2}]}$$

where $\varepsilon ={\varepsilon}^{{\prime}}+i{\varepsilon }^{{\prime}{\prime}}$ is the complex dielectric constants of the nanoparticle. It is obvious that behaves non-linearly in terms of Im{$\varepsilon$}=$\varepsilon ^{\prime\prime}$, which is responsible for optical loss, and this indicates that the relation between CD_abs_ and the loss needs a closer look. Figure 2(a) presents theoretical results of Max.{CD} versus *γ*, generally confirmed by the results of the numerical computation shown in Fig. 2(b). In this figure, columns (c) and (d) show the CD_abs_ by considering different *γ* that are determined on the curves in Fig. 2(a,b) by red and blue points. In this regard, the points A/A′ refer to the more realistic value of *γ* for gold^23^. It can be seen that by reducing the value of *γ* (corresponding to loss), the CD increases at first, and then sharply decreases. Physically, according to Eq. 3, the both terms Im{*α*} and |*α*|^2^ determine CD_abs_. Although, in the extreme case of *γ* = 0, $\varepsilon ^{\prime\prime}$ = 0, and ultimately Im{*α*} and thereby CD_abs_ diminishes. One should also consider that, by decreasing *γ*, the intensity of the point dipole oscillator (|*α*|^2^) increases and for some values, compensates the reduction of Im{*α*} in the amplitude of CD_abs_ (Eq. 3). However, by further reduction of *γ*, it declines and has a dominant role in the amplitude of CD_abs_. It is valuable to note that by decreasing *γ*, the CD_abs_ exhibits a narrower spectral line-shape. A comparison between the results of the modeling and the simulated ones confirms that the general trends are qualitatively supported by the CDE model. Indeed, the black dashed-lines in Fig. 2(c1) show *λ*_CD±_ that are calculated using the Eq. (4). Based on this equation, the optical properties of the Au, and the dimensions of the nanoblocks the resonant wavelengths of the cross hybridized modes are *λ*_CD**−**_ = 653 nm and *λ*_CD+_ = 740 nm.

### CD of the planar Au-, Ag-, and Ni-nanostructures

**Figure 3:**
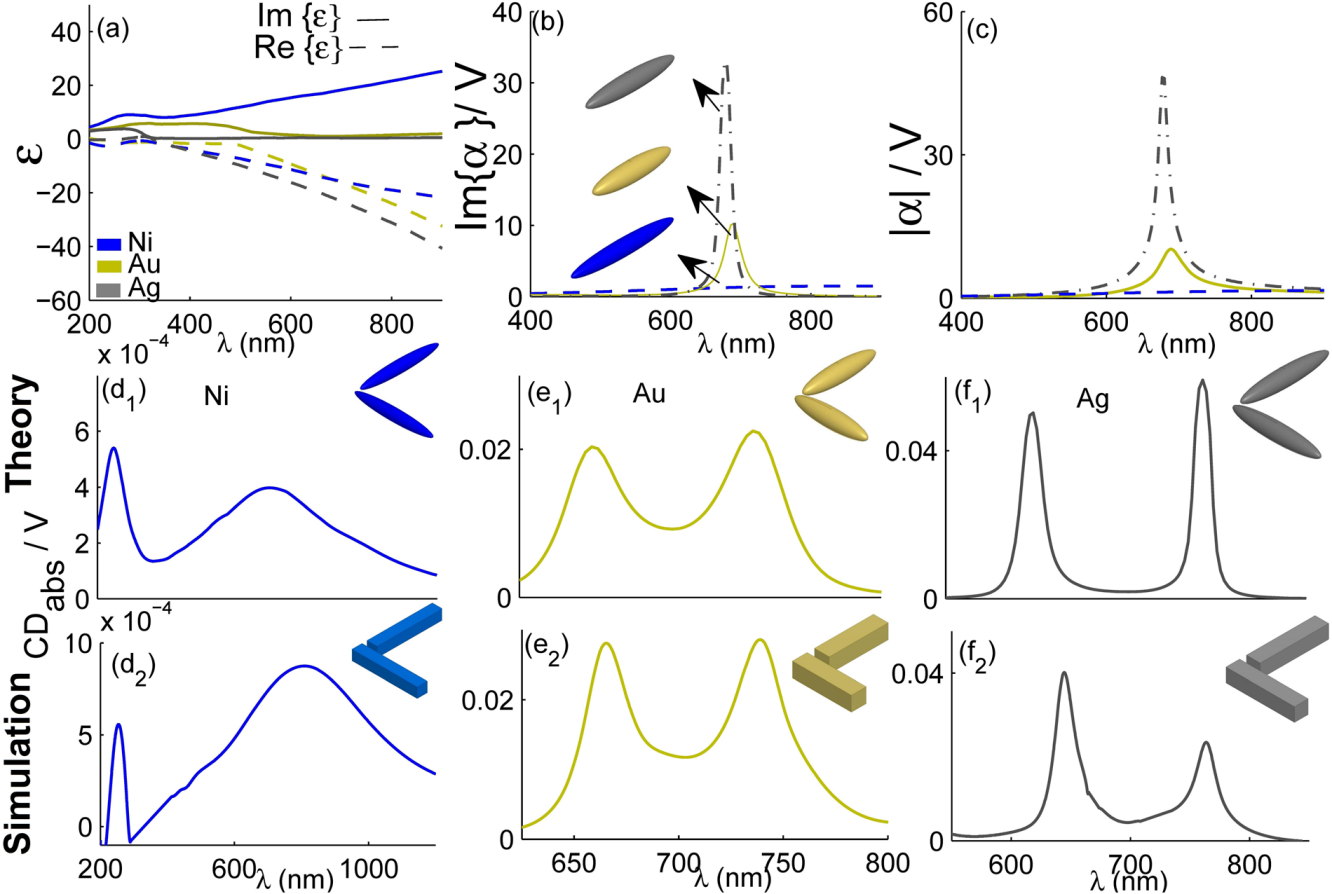
Optical properties of Au, Ag, and Ni nanoparticles. The dielectric function of the metals, normalized Im{*α*} and |*α*| of the nanospheroids, corresponding to nanoblocks with *L* = 80 nm, 100 nm, and 130 nm composed of Au, Ag, and Ni, are shown in the first-row (**a**,**b**,**c**). The theoretical results for CD_abs_ of the dimers composed of these metals shown in the second-row are compared with the corresponding numerical results in the third-row.

In order to proceed and complete the discussions, we also study CD of the plasmonic dimer composed of well-known metals with different absorption such as silver (Ag), gold (Au), and nickel (Ni), in according with ‘best-good-bad’ manner. The dielectric constants of the metals, adopted from the tabulated data in refs 26 and 27. For the assumed three pragmatic cases, the length (*L*) of the nanoparticles is defined in such a way that the LSPR, in the case of a single nanoblock, occurs nearly at the same wavelength or energy. Therefore, Au-, Ag-, and Ni-nanoparticles with lengths *L* = 80 nm, 100 nm, and 130 nm are adopted, respectively. The resonances of single ones are ~*λ* = 700 nm. Figure 3(a) indicates the real and imaginary parts of the dielectric functions of the considered metals. Figure 3(b) and (c) show the Im{*α*} and |*α*|, the two major terms of Eq. (3), for the induced dipole in the nanospheroids along the major axis, respectively. As mentioned before, the nanoblocks are modeled with prolate nanospheroids with slightly different 2*a*, which are schematically depicted in Fig. 3(b). As seen in the Fig. 3(a); although, Ni has more losses, in comparison with two other metals, and Ag has the least loss, in contrast Im{α} and |*α*| of the Ag-nanoparticle is substantial. This result marks at this point that smaller value of $\varepsilon ^{\prime\prime}$, does not always mean smaller Im{*α*} and |*α*|. Based on the indirect relation explained in Eq. 5, in these cases reduction of the Im-part of optical constant, i.e. $\varepsilon ^{\prime\prime}$, not only increases the intensity of the corresponding resonant dipole but also raises the Im-part of the resulting polarizability. Figure 3(d,e, and f) indicate the both theoretical and numerical CD for dimers made of different metals. As expected from the results of Fig. 3(b) and (c), the induced optical CD of the dimer composed of Ni is less than others; and interestingly the Ag-dimer demonstrates the highest CD among the considered cases, unrespecting to its volume. The second and third rows of Fig. 3 show the theoretical and numerical results, respectively. It can be seen there is a remarkably close correspondence between those results. It should be also noted that since the dimensions of these three dimers are not the same, *A*_xy_ may be slightly different; although, these variations are minor compared to the major effects raised from the polarizabilities, *α*_*x*_ and *α*_*y*_, removing any ambiguity. Based on these results, it can be stressed there is indirect relation between the amounts of loss ($\varepsilon ^{\prime\prime}$) and the CD intensities of single dimers made of lossy Ni, Au, and Ag. The later displays stronger CD, owing to the roles of optical properties of the single low-loss Ag-nanoparticles and their strong optical interactions in the dimer configuration.

**Figure 4:**
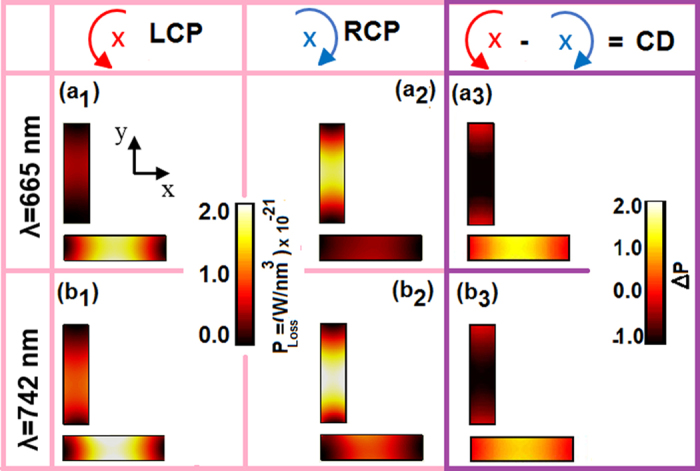
Power loss densities in the Au nanoblocks at the in-phase (*λ* = 665 nm) and out-of-phase (742 nm) hybridized LSPR modes for the RCP (a_1_,b_1_) and LCP (a_2_,b_2_) cases, respectively. The associated differences in the power loss densities (CD) are shown in (a_3_,b_3_).

To further address the difference between the optical properties of the LCP and RCP illuminations upon the considered planar plasmonic nanostructure, the power loss densities (P_Loss_) are shown at the peaks’ positions of the observed CD_abs_ spectra or the cross-hybridized LSPRs for a dimer of Au nanoblocks (Fig. 3-e_2_). According to the theory of the hybridized modes, bonding and anti-bonding, are induced in the system at *λ* = 665 and 742 nm, respectively. The power absorptions and scattering are different for the LCP and RCP incident waves. The power loss densities are shown in Fig. 4 when the structure is illuminated with RCP (a_1_, b_1_) and LCP (a_2_, b_2_) plane-waves. Interestingly, the power loss is higher at the nanoblock along the y-axis when the RCP excites the dimer; although, as seen in Figs 4(a_2_ and b_2_) it is reversed for the case of the LCP. The difference between power loss densities (∆P_Loss_) of the LCP and RCP incident waves for the hybridized modes are plotted in the right-column of Fig. 4. However the optical loss profiles computed for Ag dimer are very similar in trends (not shown) but with higher strengths (see Fig. 3f), the polarization-dependent loss distributions P_Loss_ and ∆P_Loss_ are plotted for Au nanoblocks, relying on gold’s practical considerations such as fabrication, engineering, and compatibility issues. Finally, the introduced effects and extended results provide a basic physical description of the CD phenomena in the feasible planar nanostructures which has a remarkable importance in the field of chiral spectroscopy, nanooptics, and nanophotonics.

## Conclusion

The origin of optical activity in planar metallic nanostructures was physically discussed. Based on the developed theory and the cross-hybridization model which led to the neat expressions in the quasistatic regime, it was reported that handedness dependent optical interactions influencing the absorptions are crucial and necessary for the observed optical CD in these plasmonic nanostructures. The convenient Drude model was employed to justify the theoretical predictions, and mainly to elaborate on how the loss influences the observed phenomenon. In this regard, it is straightforwardly concluded that any lossless planar dielectric or metallic structure fails to exhibit any CD, even though, they are arranged in a handedness setting with or without strongly optical interacting parts. By considering Ni, Au, and Ag as more practical cases, it was illustrated that later material exhibit stronger CD_abs_, compromising the loss effect. The presented results deepen the physical understanding and they may prompt new viable designs and related applications in the advanced and sound opto-chiral areas.

## Methods

For theoretical analysis, the nanoblocks are modeled by the prolate nanospheroids and the electric polarizabilities are calculated using the semi-analytical formula for the polarizability of prolate^28^:

6 $$\alpha (\omega )=\frac{V}{4\pi }\frac{(\varepsilon -{\varepsilon }_{m})}{{\varepsilon }_{m}+({L}_{x}-i\frac{{k}^{3}V}{6\pi }-\frac{{k}^{2}V}{4\pi }{D}_{x})(\varepsilon -{\varepsilon }_{m})}$$

which *V* = 4π*abc*/3 is the particle volume. *ε* and *ε*_m_ are the dielectric coefficients of the nanoparticle and the surrounding medium, respectively. The parameters *L*_x_ and *D*_x_ are the static and dynamic depolarization factors of the prolate along the x-axis. Using the CDE (Eq. 1), the modified polarizabilities are obtained as:

7 $${P}_{{\rm{i}}}=\frac{{E}_{\text{inc},i}+{A}_{12}{\alpha }_{{\rm{i}}}{E}_{\text{inc},j}}{1-{({\alpha }_{{\rm{i}}}{A}_{12})}^{2}}\,\,\,\,i,j=x,y$$

substitution Eq. (7) into Eq. (2) for LCP and RCP incident fields individually, considering $d{\rm{\Omega }}={\sin }^{2}(\theta )d\theta \,d\varphi$,$\exp (-ik\hat{{\bf{n}}}.{{\bf{r}}}_{{\rm{j}}})\approx 1-ik\hat{{\bf{n}}}.{{\bf{r}}}_{{\rm{j}}}$, and obtaining Eq. (2) on the entire space $0 < \theta \, < \pi ,$ ($0 < \phi \, < 2\pi$), C_abs_ is calculated. The difference between C_abs_ for LCP and RCP light result in Eq. (3). In the simulation procedure, we use the Finite Integration Technique (FIT). The results are obtained for isolated dimers in the simulation volume which is set to the λ_0_/4 (a quarter of the resonant wavelength of a single nanorod). Tetrahedral meshes with cell size within 1–6 nm are applied in the dimer region. The perfectly matched layer (PML) boundary condition with the reflection level < 0.0001 is used to model the free space.
